# Association of Urban Slum Residency with Infant Mortality and Child Stunting in Low and Middle Income Countries

**DOI:** 10.1155/2013/604974

**Published:** 2013-09-17

**Authors:** Hmwe Hmwe Kyu, Harry S. Shannon, Katholiki Georgiades, Michael H. Boyle

**Affiliations:** ^1^Department of Psychiatry and Behavioural Neurosciences and Offord Centre for Child Studies, Hamilton Health Sciences, McMaster University, 1280 Main Street West, Hamilton, ON, Canada L8S 4L8; ^2^Department of Clinical Epidemiology & Biostatistics, Faculty of Health Sciences, McMaster University, 1280 Main Street West, Hamilton, ON, Canada L8S 4L8

## Abstract

This study aimed to (i) examine the contextual influences of urban slum residency on infant mortality and child stunting over and above individual and household characteristics and (ii) identify factors that might modify any adverse effects. We obtained data from Demographic and Health Surveys conducted in 45 countries between 2000 and 2009. The respondents were women (15–49 years) and their children (0–59 months). Results showed that living in a slum neighborhood was associated with infant mortality (OR = 1.34, 95% CI = 1.15–1.57) irrespective of individual and household characteristics and this risk was attenuated among children born to women who had received antenatal care from a health professional (OR = 0.79, 95% CI = 0.63–0.99). Results also indicated that increasing child age exacerbated the risk for stunting associated with slum residency (OR = 1.19, 95% CI = 1.16–1.23). The findings suggest that improving material circumstances in urban slums at the neighborhood level as well as increasing antenatal care coverage among women living in these neighborhoods could help reduce infant mortality and stunted child growth. The cumulative impact of long-term exposure to slum neighborhoods on child stunting should be corroborated by future studies.

## 1. Introduction

More than 800 million people (32.7% of urban populations) resided in urban slums in developing countries in 2010 [1]. A slum household is defined as “a household lacking one or more of the following conditions (indicators): improved water; improved sanitation; sufficient living area; durable housing; and secure tenure” [1]. Over the period from 2000 to 2010, the population living in slums increased by 61 million. This increase mainly occurred in Sub-Saharan Africa, Southeastern Asia, and Western Asia [1].

Slum dwellers experience considerably higher levels of socioeconomic disadvantage than other urban residents. However, not all families living in slums are poor or uneducated. For example, a survey conducted in 211 slums in India reported that the occupations of slum dwellers vary from sweepers and vendors to government employees and small entrepreneurs and there were even a few computer professionals, teachers, nurses, and doctors living in some slums [2]. People with “adequate or good” income may choose to live in a slum especially if they have businesses in or around the slum [2]. Previous studies reported that malnutrition or mortality rates of children living in urban slums are higher than those of rural children or the overall urban population [3, 4], but these are crude estimates unadjusted for socio-economic status and other characteristics of resident families. An important question remains unanswered: is this increased risk attributable to the socio-economic status of the slum residents (compositional effect) or the environmental characteristics of the slum neighborhood itself (contextual effect)? If an adverse effect associated with living in a slum neighborhood persists after adjusting for characteristics of inhabitants, the next step is to identify factors that could attenuate or exacerbate this effect on child health.

Higher socio-economic status (e.g., household wealth) can improve child health by providing an opportunity to improve the material circumstances of the family and to purchase goods and services that are beneficial to health [5]. Higher levels of maternal education can influence child survival through better health care practices regarding nutrition, hygiene, and prevention and treatment of diseases [6]. As a result, the effect of residing in a slum neighborhood on child health can be accounted for or modified by household wealth and maternal education. Timaeus and Lush [7] illustrated this using data from Egypt and Brazil. In Egypt, children living in better-off families with area access to improved drinking water and sanitation had lower mortality rates, while in Brazil, children living in worse-off families exposed to poor sanitation had higher mortality rates. However, that analysis was neither adjusted for family circumstances nor tested for their moderating influence (statistical interactions), leaving open the question of attribution, the extent to which slum residency effects might be attributable to other important variables. 

Interventions during the earliest periods of life (e.g., prenatal period or infancy) may also modify slum effects. According to the World Health Organization (WHO) [8], these early interventions (e.g., supplementary feeding of pregnant women and intervention to control diseases) can have their largest influence on the physical growth of young children. Antenatal care may provide such health benefits or improve child survival by identifying and treating conditions that are harmful to the health of the newborn and/or the mother and by educating pregnant women on safe motherhood [9]. 

### 1.1. Mechanisms through Which Slum Residency Could Influence Child Mortality and Stunting

Available evidence suggests that causes of death in urban slum children include poor neonatal care [3], neonatal tetanus [10, 11], diarrhea, and respiratory infections [3, 12]. While tetanus immunization (routinely provided during antenatal care) and clean deliveries could prevent neonatal tetanus [13], rates of antenatal care or institutional deliveries are usually much lower in urban slums than nonslum urban areas [14–16]. Moreover, lack of access to clean water and sanitation facilities could create a fecally contaminated environment (e.g., Ahmedabad slums in India) [3], which could lead to diarrhea-related mortality [17] or repeated episodes of diarrhea resulting in stunting [18], an indicator of long-term malnutrition in children [19]. Furthermore, an earth floor (a characteristic of many slum households) can be a breeding ground for various infectious agents [20], and living in earth floor houses has been associated with increased risk of diarrhea [21] and being a carrier of Streptococcus pneumoniae [22], a bacteria that commonly causes acute respiratory infections in children. Taken together, it is reasonable to expect increased risk of mortality and morbidity in children living in an infectious or contaminated neighborhood irrespective of household-level access to water and sanitation facilities. Among children who survive infectious diseases, one may also expect increased risk for stunting especially among older children (or those with longer duration of stay) in a slum neighborhood. Our analysis explores these hypotheses.

### 1.2. Study Objectives and Rationale

The objectives of this study were (i) to examine the contextual influences of urban slum residency on infant mortality and child stunting over and above individual and household characteristics and (ii) to determine if household wealth, maternal education, antenatal care, and child age modify the adverse effects associated with urban slum residency. 

 This study offers several important advantages over previous work. First, the definition and measurement of slum residency are based on a set of commonly defined slum indicators recommended by the United Nations. Second, the analysis is designed (a) to disaggregate the effects of residency in a slum neighborhood (contextual effect) versus living in particular households (compositional effect) and (b) to test the moderating influences of one (family/household variables) on the other (exposure to slum environments). Third, the study draws information from 45 countries, making it uniquely able to test for the consistency of slum residency effects across many countries. Fourth, the study tests for an interaction between slum residency and child age in their effects on stunting to determine if length of exposure to slum residency is associated with an exaggerated adverse effect on child health. 

## 2. Method

We obtained data from nationally representative Demographic Health Surveys (DHS) conducted in 45 low and middle income countries between 2000 and 2009. Study details are available at the DHS website (http://www.measuredhs.com/aboutdhs/) [23] and we provide a brief outline here. The sampling frame is a list of nonoverlapping area units (enumeration areas) that cover the entire country and serve as the primary sampling units or clusters. In the selected clusters, all households are listed and a fixed proportion is chosen by systematic sampling. All eligible persons in selected households are included in the final sample. DHS conducts interviews with all women aged 15–49 years in these households, assesses their children (0–59 months), and measures maternal and child heights using a standardized approach. Child height data are available in only 43 countries. In some countries, height data are collected in a random subset (one-third or half) of the sample. The response rates vary from 92% in Ukraine to 98% in Cambodia.

### 2.1. Concepts and Measures

#### 2.1.1. Dependent Variables

*Infant Mortality*. This is a binary variable that indicates whether a child born in the 5 years before the survey survived up to 12 months of age. For all biological children born in the previous 5 years, DHS collected information from mothers about the timing of these births and their survival status (alive or dead) at the time of interview. If alive, mothers were asked the age of their child; if dead, mothers were asked the child's age at death. The survival status of the child at one year of age was determined from this information.

*Stunting*. Height measurements for children (0–59 months) of sampled women were taken using a standardized measuring board. Children younger than 24 months were measured lying down on the board, whereas standing height was measured for older children. The height measures were converted to standard deviation units (*z* scores) using the World Health Organization (WHO) Child Growth Standards [24]. They were then classified as no stunting (*z* ≥ −2 standard deviation (SD)) and stunting (*z* < −2 SD) [25].

#### 2.1.2. Measuring Slum Household and Slum Neighborhood

Developing a common measure of slum residency poses some challenges. Available evidence suggests that two components of the UN slum definition may be unsuitable indicators in a cross-national study. “Secure tenure” is not easy to measure or monitor because it carries a legal meaning [1], and information on tenure is not available for most countries. Furthermore, sufficient living area, defined as not more than 3 household members sharing the same room [1], could misclassify a nonslum household as a slum household in many cultures where “cosleeping” with children is the norm [26].

*Slum Household*. In this study, a slum household was defined as a household located in an urban area and lacking *at least two *of the following indicators: *access to improved water* (e.g., piped connection to house or plot, public tap/stand pipe, tube well/bore hole, and protected dug well) [1], *access to improved sanitation* (e.g., flush or pour flush to piped sewer system, septic tank or pit latrine, and ventilated improved pit latrine) [1], and *structural quality/durability of dwellings* (i.e., whether the structural quality of floor, wall, or roof of a household is natural, rudimentary, or finished) [27]. Information concerning wall and roof is not available for many countries. In this study a house is considered not durable if the floor of the house was made of natural or rudimentary materials (i.e., earth, mud, sand, wood planks, palm, and bamboo).

*Slum Neighborhood*. UN-Habitat [28] recommended the use of enumeration areas to localize slum neighborhoods as these areas represent the “smallest household aggregation” in many countries and are relatively homogeneous. To construct the slum neighborhood variable for this study, slum households were aggregated up to the enumeration area or cluster level. Using the threshold of 50% recommended by UN-Habitat [28], a cluster is defined as a slum neighborhood if it includes 50% or more slum households. 

#### 2.1.3. Covariates

We selected covariates to adjust for possible confounding in the analyses (control variables) and to examine the potential of selected variables to modify associations between slum residency and infant mortality or child stunting (i.e., household wealth, maternal education, and antenatal care for infant mortality, with the addition of child age for stunting). The following variables served as covariates: GDP, household wealth (wealth index), antenatal care, maternal height for age, place of delivery, breastfeeding, mother's education (total years of schooling), maternal age at birth of child, birth order, previous birth interval, child gender, multiple births, child age in months (rescaled for the purposes of the analyses so that one unit increase represents one year) and duration of residence.

*GDP*. At the national level, GDP is gross domestic product per capita converted to constant 2005 international dollars based on purchasing power parity [29]. It has been rescaled for the purposes of analyses so that one unit increase in GDP represents 1000 international dollars.

*Wealth Index*. We used the *wealth index* [30, 31] variable already available in each DHS dataset. It has been shown to be a good proxy for long-run household wealth [31, 32]. It was derived from an index (generated through principal component analysis) of household assets ranging from televisions to bicycles and characteristics of the dwelling depending on the specific questions asked in each country. The index was standardized to a mean of 0 and a standard deviation of 1 and higher scores refer to greater wealth.

*Antenatal Care*. Women were asked whether they had received antenatal consultation from a health professional (doctor, nurse, or midwife) during the antenatal period. They were also asked about the total number of antenatal visits during pregnancy. For women who had received at least one antenatal consultation from a health professional, two dummy variables were created and coded 1 if applicable: “1–3 antenatal visits” and “4 or more antenatal visits.” The reference group coded 0 was women who did not receive any antenatal consultation from a health professional.

*Maternal Height for Age*. DHS measured maternal heights using a standardized measuring board and calculated height for age standard deviations from the reference median based on the CDC standard deviation-derived growth reference curves [27].

*Place of Delivery*. For each child born in the previous 5 years before the survey, respondents were asked whether (1) a health professional (doctor, nurse or midwife) was present at delivery and (2) whether the birth occurred at home or in a health facility. Two dummy variables were created and coded as 1 if applicable: “assisted delivery by a health professional at home” and “delivery at a health facility.” The reference category coded 0 was delivery at home with no skilled assistance.

*Breastfeeding*. For each child born in the previous 5 years before the survey, respondents were asked whether the child was ever breastfed. Women who had ever breastfed their babies were asked how long after birth they first put the baby to the breast. In some countries, the latter question was asked only for the most recent births. For the analyses, three dummy variables were created and coded as 1 if applicable: “early initiation of breastfeeding” (within an hour after birth) [33], “unknown timing of breastfeeding initiation,” and “never breastfed.” The reference category code 0 was “delayed initiation of breastfeeding” (more than one hour after birth).

### 2.2. Sample for Analysis

For this study, we identified DHS conducted in 53 countries between 2000 and 2009. Eight out of the 53 countries were excluded for the following reasons: information on duration of stay at the current place of residence was not available (4 countries); specific information on toilet facility was not available to differentiate between improved and unimproved sanitation (2 countries); and the possibility of a nonrepresentative sample because there were massive slum clearance projects around the DHS survey periods (2 countries). The following 45 countries with requisite data were included: Armenia, Albania, Azerbaijan, Bangladesh, Benin, Bolivia, Burkina Faso, Cambodia, Cameroon, Congo-Brazzaville, Democratic Republic of the Congo, Colombia, Dominican Republic, Ethiopia, Gabon, Ghana, Guinea, Haiti, Honduras, India, Jordan, Kenya, Lesotho, Liberia, Madagascar, Mali, Malawi, Moldova, Namibia, Nepal, Nicaragua, Niger, Nigeria, Philippines, Rwanda, Sao Tome and Principe, Senegal, Sierra Leone, Swaziland, Tanzania, Timor-Leste, Turkey, Uganda, Ukraine, and Zambia. 

Only the usual residents (not visitors) of the households located in urban areas of the 45 countries (*n* = 128,023) were eligible to be included in this study. About 2% of children had missing information on study variables and an additional 5% of children were missing data on covariates and were excluded. The sample for analysis included 118,598 children from 45 countries for the outcome infant mortality, and 83,145 children from 43 countries for the outcome stunting (child height data were not available in Philippines and Ukraine).

### 2.3. Data Analysis

Multilevel logistic regression models were constructed using the statistical software MLwiN version 2.26 [34] to conduct a three-level analysis with children nested in clusters (neighborhoods) which are nested in countries. A sample multilevel logistic regression model used to estimate the association between slum residency and infant mortality is presented as follows:

(1) log⁡(yijk)=β0jk+β1  SlumNeighborhoodjk +  β2  SlumHouseholdijk+β3GDPk +⋯+v0k+u0jk.

In this model, the dependent variable *y*_*ijk*_ represents the odds of a child *i* in neighborhood *j* and country *k* dying in the first year of life. *β*_0*jk*_ represents the fixed effect intercept (i.e., the log-odds of infant mortality when all variables in the model equal zero). The *β* coefficients (*β*_1_, *β*_2_, etc.) quantify the strength of association of the dependent variable with independent and control variables measured throughout the hierarchy of levels. These *β* coefficients are log odds ratios which were exponentiated into odds ratios. All variables and interaction terms included in this model are presented in Table 2. This model also illustrates the disaggregation of “contextual” and “compositional” effects. For example, SlumNeighborhood_*jk*_ and GDP_*k*_ are contextual variables at the neighborhood and country levels, respectively, while SlumHousehold_*ijk*_ is a compositional or individual-level variable. Random effects estimates *v*_0*k*_ and *u*_0*jk*_ were included in the model to allow for between-country and between-neighborhood variability in infant mortality. In this study, household-level variables were treated as child-level variables because the average number of children (aged 0–59 months) clustered in households (1.4) was too small to have a separate level. All continuous variables were grand-mean centered.

Information regarding antenatal care was collected only for the most recent births in 43 countries from a random sample of households (DHS deliberately refrained from collecting this information for the older children in these households) and maternal height data were collected in only 42 countries. According to Acock [35], data that are missing as a result of valid skips should not be imputed. For the analyses, we created two dummy variables to indicate if information on these variables was available. 

The models begin by examining the effects of living in a slum neighborhood (after adjusting for residing in a slum household) on the outcome variables. Next, all covariates were included in Model 2 to examine if a statistically significant adverse effect of slum neighborhood persisted after adjusting for these variables. If such an effect was present, we included interaction terms in Model 3 to identify factors that might modify (attenuate or exacerbate) the adverse effect of living in a slum neighborhood. For the outcome stunting, we applied the same decision rule for testing interactions, with one exception. Irrespective of the association between living in a slum neighborhood and child stunting, we tested for an interaction between slum neighborhood and child age in Model 3. Assuming that older versus younger children have longer exposures to the adverse effects of living in a slum neighborhood, we hypothesized that older children would be at exaggerated risk for stunting. 

We conducted two sensitivity analyses by (1) controlling for survey year dummy variables (to ensure that the survey year does not have any influence on the association of slum residency with infant mortality and stunting) and (2) defining a slum household using a lower threshold definition (i.e., lacking one or more of the following indicators: improved water, sanitation, or durable housing).

## 3. Results

 There were 118,598 children living in 13,054 clusters in urban areas from 45 countries. The average numbers of children and households per cluster were 9.1 and 6.5, respectively. About 12% of neighborhoods were slum neighborhoods and about 18% of children lived in slum households (data not shown in table).  Table 1 presents sample characteristics in slum and non-slum neighborhoods and the overall sample. Infant mortality was 4.6% in slum neighborhoods versus 3.0% in non-slum neighborhoods while child stunting was 36.8% versus 24.1%, respectively. Slum residents were about three times more likely to report having no antenatal care compared with non-slum residents (17.9% versus 6.0%). There was substantial between-country variation in the proportion of women who did not receive antenatal care in slum neighborhoods (0% in Ukraine versus 53.6% in Mali) (data not shown). 

Table 2 presents odds ratios (OR) for infant mortality associated with slum residency after adjusting for covariates. In Model 1, residing in a slum neighborhood was associated with infant mortality after adjusting for living in a slum household (OR = 1.30, 95% CI = 1.17–1.45). In Model 2, the slum neighborhood effect was still statistically significant (OR = 1.20, 95% CI = 1.07–1.35) after including the covariates. In Model 3, there was a statistically significant interaction between slum neighborhood and 1–3 antenatal visits (OR = 0.79, 95% CI = 0.63–0.99). This interaction implies that the risk for infant mortality associated with residing in a slum neighborhood was attenuated or muted among children born to women who had 1–3 antenatal visits. However, there were no statistically significant interactions involving household wealth or maternal education. To determine if there was between-country variation in the association between slum neighborhood and infant mortality, we specified slum neighborhood to have a random slope in a separate analysis. The test for random slope was not statistically significant, suggesting that the slum neighborhood effect on infant mortality was consistent across countries.

Table 3 presents ORs for child stunting associated with slum residency. In Model 1, residing in a slum neighborhood was associated with stunting after adjusting for living in a slum household (OR = 1.28, 95% CI = 1.20–1.37). In Model 2, the association was no longer statistically significant after including covariates. In Model 3, there was a statistically significant interaction between slum neighborhood and child age on stunting (OR = 1.19, 95% CI = 1.16–1.23). This interaction implies that increasing child age elevated the risk for stunting associated with slum residency. 

The sensitivity analysis controlling for survey year dummy variables did not alter the main analysis results. The sensitivity analysis (data not shown) using a lower threshold slum definition (i.e., one or more indicators) yielded the following adjusted estimate (Model 3) for the association between slum neighborhood and stunting (OR = 1.08, 95% CI = 1.03–1.14). Similar to the primary analysis, there was a significant interaction between child age and slum neighborhood (OR = 1.12, 95% CI = 1.08–1.14). The adjusted association between slum neighborhood and infant mortality (OR = 1.07, 95% CI = 0.97–1.18) (Model 2) did not reach statistical significance in the sensitivity analysis (data not shown).

## 4. Discussion

 This study is the first to cross nationally examine the contextual influences of living in a slum neighborhood on child health using a common measure of slum residency. We did so by attempting to disaggregate the effect on child health of living in a slum neighborhood (contextual effect) versus living in disadvantaged circumstances defined by individual and household characteristics (compositional effects). In this study, living in a slum neighborhood was associated with infant mortality irrespective of individual and household characteristics and this association was consistent across countries. In other words, children from both very poor and better-off households located in a slum neighborhood were at increased risk for infant mortality. This increased risk was similar for children living in the 45 different countries included in the analysis and was attenuated among children born to women who had received antenatal care from a health professional. Results also showed that increasing child age exacerbated the risk for stunting associated with residing in a slum neighborhood. 

In this study, about 12% of neighborhoods were slum neighborhoods and about 18% of children lived in slum households. When using a lower threshold definition for a slum household, the corresponding prevalence estimates for slum neighborhood and children living in slum households are 56% and 55%, respectively. These estimates may not be directly comparable to those of the United Nations because the latter reports the overall slum population as a percentage of urban residents: it does not specifically report the percentage of slum neighborhoods or children living in slum households. In addition, the United Nations estimates are not based on a uniform number of indicators: only two indicators (e.g., water and sanitation only) in some countries and three or four indicators in others [36]. In this study, we selected countries based on the availability of information on all three indicators: water, sanitation and housing condition and used a common approach to define a slum household and a slum neighborhood.

Our study adds to concerns raised by Timaeus and Lush [7] about the harmful effects of poor environmental conditions on child health. We also examined if the adverse effects of urban slum residency on the outcomes were modified by certain individual and household characteristics including child age, antenatal care, household wealth and maternal education. We found that increasing child age elevated the adverse effects of residing in a slum neighborhood on child stunting. Prolonged exposure might account for this in older children although we cannot be sure due to the cross-sectional nature of the study. Given that stunting in early childhood can result in irreversible negative consequences (e.g., delayed motor development, impaired cognitive function, and poor school performance) [37], a more complete understanding of the long-term impact of exposures to slum environments on child stunting is warranted. Future studies should examine this association longitudinally to establish causal relations.

Our findings also indicated that antenatal care (1–3 visits) attenuated the adverse effects of slum residency on infant mortality. We did not find any modifying influences of (i.e., interactions with) antenatal care (4 or more visits), household wealth, and maternal education. This simply means that the beneficial main effects (e.g., reduction in risk for infant mortality) observed for household wealth, maternal education, and 4 or more antenatal care visits were similar for both slum residents and nonresidents. 

Rose and colleagues [38] have distinguished between “protective factors” and “resource factors”—the former exerting beneficial effects under conditions of risk and the latter exerting beneficial effects generally. Because there are different levels of antenatal care (e.g., frequency of visits), it is possible for antenatal care to serve as both a protective factor and a resource factor if these different levels of care serve different functions (i.e., at one level, reducing child health problems only for those residing in urban slums, and at another level, reducing child health problems in all children regardless of their residency risk (living in slums versus non-slums). The World Health Organization [9] recommended that women have at least four antenatal visits but the results of our study show that even 1–3 antenatal visits may be a protective factor for infant mortality under conditions of risk. While causes of death in children living in urban slums include poor neonatal care [3], neonatal tetanus [10, 11], diarrhea, and respiratory infections [3, 12], the potential benefits of antenatal care (e.g., providing tetanus immunization, identifying and treating harmful health conditions of mothers and newborns, and educating mothers on healthy pregnancy/delivery) [9, 13] might possibly contribute to this protective effect. Receiving 4 or more antenatal visits appears to reduce the risk of infant mortality equally among slum residents and nonresidents and may therefore be regarded as a resource factor. Similarly, household wealth and maternal education are more likely to be resource factors for preventing infant mortality in this study. 

This study has some limitations. First, newly emerging slum neighborhoods excluded in the census enumeration might have been omitted during DHS data collection. If that was the case, our findings may not be representative of all slum settlements in the included countries. Second, because countries may vary in their approach to defining slums, a uniform definition may not capture all slums across countries given the wide disparities of socioeconomic and physical environmental conditions. However, most slum definitions [39, 40] share common characteristics (i.e., water, sanitation, and housing conditions) that were used to construct the slum variable in this study. In this study, a lower threshold definition of slums leads to weaker infant mortality effects in the same direction. Given that some slum indicators are not stand-alone, using the lower threshold definition may produce an overestimation of slum households as well as the observed weaker association between slum residency and infant mortality. Third, there appears to be some overlap in the items (e.g., water and sanitation facilities) used to define a slum household and those used to define the household wealth variable. However, since the purposes of constructing the two variables are different, the way the items are defined is not the same. For example, for the purposes of constructing the household wealth variable, toilet facility is classified as having a flush toilet (yes, no). For the purposes of constructing a slum household variable, a flush toilet is defined as improved or unimproved depending on whether it is connected to a sewer system. The point-biserial correlation between the two variables (slum household and household wealth) is −0.206 (*P* < 0.001). This conservative approach allocates maximal weight for the effect of household wealth. Although controlling for household wealth reduced the slum residency effects, there was still a statistically significant and meaningful adverse effect on child mortality. Fourth, the exact duration of residence in slum neighborhoods was not known but 95% of the respondents reported to have lived at their current places of residence for at least one year at the time of the survey. Finally, observational studies such as the present study are inevitably vulnerable to residual confounding and omitted variable biases. Although we have done our best to control for all possible confounding variables available in the DHS, we acknowledge that residual confounding remains a plausible explanation for our results.

The large diverse sample, representing 45 low and middle income countries, is a strength of this study, making it possible to estimate the association between slum residency and child health cross nationally and to test the extent to which this association varies from country to country. The consistency of the adverse effects of residing in a slum neighborhood on both outcomes and the consistent direction of adverse effects in the sensitivity analysis using a lower threshold definition support the robustness of our findings. 

## 5. Conclusions

 The findings of this study indicate the importance of contextual influences of urban slum residency at the neighborhood level on child health. Living in a slum neighborhood was associated with infant mortality irrespective of the socio-economic status and other characteristics of households and families. Results also showed that the risk of stunting in slum neighborhoods was greater for older children. The impact of long-term exposure to slum neighborhoods on child stunting should be corroborated by future studies. Maternal use of antenatal care services might alleviate the harmful effect of slum residency on infant mortality but antenatal care coverage among slum women remains low. Improving the material circumstances in urban slums at the neighborhood level as well as increasing antenatal care coverage among women living in these neighborhoods could help reduce stunted child growth and infant mortality associated with urban slum residency.

## Figures and Tables

**Table 1:** Sample characteristics in urban slum and nonslum neighborhoods in 45 countries^a^.

	Slum neighborhood (*N* = 21,305)	Nonslum neighborhood (*N* = 97,293)	Overall sample (*N* = 118,598)
Outcomes (%)			
Infant mortality	4.6	3.0	3.3
Stunting^b^	36.8	24.1	26.0
Variables			
Household wealth mean (SD)	−0.04 (1.9)	0.8 (2.6)	0.7 (2.5)
Household wealth category (%)			
Poorest	14.9	4.4	6.0
Poorer	19.2	9.4	10.9
Middle	25.2	17.1	18.3
Richer	25.7	27.9	27.6
Richest	15.1	41.1	37.2
Maternal education (years) mean (SD)	4.3 (4.1)	7.8 (4.9)	7.3 (4.9)
Maternal education category (%)			
No education	35.2	15.7	18.6
Primary	39.6	25.7	27.8
Secondary	23.3	44.8	41.5
Higher than secondary	1.9	13.8	12.0
Maternal height-for-age mean (SD)	−1.3 (1.2)	−1.1 (1.2)	−1.2 (1.2)
Antenatal care (%)			
None	17.9	6.0	7.6
1–3 visits	28.0	17.5	19.0
Four or more visits	54.1	76.5	73.4
Place of delivery (%)			
At home and no skilled assistance	34.4	12.0	15.4
At home with skilled assistance	4.3	3.8	3.9
At health facility	61.3	84.2	80.7
Birth order (%)			
First born	23.7	31.8	30.6
2-3 born	36.0	42.1	41.1
4-5 born	21.6	16.2	17.0
6+ born	18.7	9.9	11.2
Previous birth interval (%)			
<19 mths	7.3	7.1	7.1
19–35 mths	34.1	26.1	27.3
≥35 mths	34.9	35.0	35.0
Maternal age at child birth (%)			
<20 years	18.1	15.2	15.6
20–24 years	29.8	31.0	30.8
25–29 years	23.2	26.6	26.1
30–34 years	16.1	16.6	16.5
≥35 years	12.9	10.7	11.0
Male child (%)	51.2	51.4	51.4
Multiple births (%)	2.3	2.2	2.2
Duration of residence (%)			
<4 years	21.7	26.8	26.0
5–14 years	27.3	28.8	28.5
15 years or more	51.0	44.5	45.5
Breastfeeding (%)			
Never breastfed	4.3	5.5	5.4
Early	52.9	48.4	49.1
Delayed	29.7	31.3	31.1
Unknown timing	13.1	14.7	14.5

^a^Note: sample sizes were unweighted. Means, SDs, and % were weighted.

^b^Nonslum neighborhood (*N* = 68,391), slum neighborhood (*N* = 14,754), overall sample (*N* = 83,145).

**Table 2:** Odds ratios and 95% CIs for infant mortality associated with slum residency after adjusting for covariates.

	Model 1 OR (95% CI)	Model 2 OR (95% CI)	Model 3 OR (95% CI)
Intercept (SE)	−3.55 (0.08)***	−3.71 (0.13)***	−3.75 (0.13)***
*Study variables *			
Level 2 (Cluster)			
Nonslum neighborhood	Ref.	Ref.	Ref.
Slum neighborhood	1.30 (1.17–1.45)***	1.20 (1.07–1.35)**	1.34 (1.15–1.57)***
Level 1 (Child)			
Nonslum household	Ref.	Ref.	Ref.
Slum household	1.25 (1.14–1.37)***	1.07 (0.97–1.19)	1.07 (0.97–1.19)
*Covariates *			
Level 3 (country)			
GDP rescale		0.90 (0.85–0.96)**	0.90 (0.85–0.96)**
Level 1 (child)			
Household wealth		0.95 (0.93–0.97)***	0.95 (0.93–0.98)***
Maternal education (years)		0.97 (0.96–0.98)***	0.97 (0.96–0.98)***
Maternal height		0.93 (0.90–0.97)***	0.93 (0.90–0.97)***
Maternal height (missing data)		1.13 (1.00–1.27)	1.13 (1.00–1.27)
Antenatal care			
No		Ref.	Ref.
1 to 3 visits		0.80 (0.69–0.93)**	0.86 (0.73–1.02)
Four or more visits		0.70 (0.61–0.81)***	0.74 (0.63–0.85)***
Antenatal care (missing)			
No		Ref.	Ref.
Yes		1.39 (1.19–1.61)***	1.39 (1.20–1.62)***
Place of delivery			
At home and no skilled assistance		Ref.	Ref.
At home with skilled assistance		0.86 (0.72–1.03)	0.87 (0.72–1.04)
At health facility		0.91 (0.82–1.00)	0.91 (0.82–1.00)
Birth order			
First born		Ref.	Ref.
2-3 born		0.90 (0.80–0.99)*	0.89 (0.80–0.99)*
4-5 born		1.04 (0.91–1.20)	1.04 (0.91–1.20)
6+ born		1.11 (0.94–1.31)	1.11 (0.94–1.31)
Previous birth interval			
19–35 mths		Ref.	Ref.
<19 mths		1.67 (1.48–1.89)***	1.67 (1.48–1.89)***
≥35 mths		0.94 (0.85–1.03)	0.94 (0.85–1.03)
Maternal age at child birth			
<20 years		Ref.	Ref.
20–24 years		0.86 (0.77–0.97)*	0.87 (0.77–0.97)*
25–29 years		0.87 (0.77–0.99)*	0.88 (0.77–0.99)*
30–34 years		0.90 (0.78–1.05)	0.91 (0.78–1.05)
≥35 years		1.13 (0.95–1.34)	1.13 (0.95–1.34)
Male child			
No		Ref.	Ref.
Yes		1.21 (1.13–1.30)***	1.21 (1.13–1.30)***
Multiple birth			
Singleton		Ref.	Ref.
Multiple birth		3.16 (2.75–3.64)***	3.18 (2.76–3.66)***
Duration of residence			
≥15 years		Ref.	Ref.
<4 years		0.55 (0.50–0.61)***	0.55 (0.50–0.61)***
5–14 years		0.91 (0.83–0.99)*	0.91 (0.84–0.99)*
Breastfeeding			
Delayed		Ref.	Ref.
Early		0.99 (0.91–1.10)	0.99 (0.91–1.10)
Unknown timing		1.21 (1.04–1.39)*	1.21 (1.04–1.39)*
Never breastfed		21.24 (19.07–23.66)***	21.29 (19.11–23.71)***
Interactions			
Slum neighborhood∗ANC 1 to 3 visits			0.79 (0.63–0.99)*
Slum neighborhood∗ANC ≥4 visits			0.84 (0.69–1.03)
Slum neighborhood∗household wealth			0.99 (0.95–1.04)
Slum neighborhood∗maternal education			1.01 (0.99–1.03)

Note: level 2 and level 3 variables are contextual variables. Level 1 variables are compositional variables.

*P < 0.05, **P < 0.01, ***P < 0.001.

**Table 3:** Odds ratios and 95% CIs for child stunting associated with slum residency after adjusting for covariates.

	Model 1 OR (95% CI)	Model 2 OR (95% CI)	Model 3 OR (95% CI)
Intercept (SE)	−1.25 (0.08)***	−1.21 (0.09)***	−1.19 (0.09)***
*Study variables *			
Level 2 (Cluster)			
Nonslum neighborhood	Ref.	Ref.	Ref.
Slum neighborhood	1.28 (1.20–1.37)***	1.05 (0.98–1.12)	1.04 (0.97–1.10)
Level 1 (Child)			
Nonslum household	Ref.	Ref.	Ref.
Slum household	1.50 (1.42–1.58)***	1.13 (1.07–1.19)***	1.13 (1.08–1.20)***
*Covariates *			
Level 3 (country)			
GDP rescale		0.91 (0.87–0.96)***	0.91 (0.87–0.96)***
Level 1 (child)			
Household wealth		0.91 (0.90–0.92)***	0.91 (0.90–0.92)***
Maternal education (years)		0.96 (0.96–0.96)***	0.96 (0.96–0.96)***
Maternal height		0.70 (0.69–0.71)***	0.70 (0.69–0.71)***
Maternal height (missing data)		1.33 (1.12–1.58)***	1.33 (1.12–1.58)***
Antenatal care			
No		Ref.	Ref.
1 to 3 visits		0.88 (0.82–0.95)***	0.86 (0.80–0.93)***
Four or more visits		0.82 (0.76–0.88)***	0.80 (0.75–0.86)***
Antenatal care (missing data)			
No		Ref.	Ref.
Yes		0.78 (0.71–0.86)***	0.73 (0.67–0.81)***
Place of delivery			
At home and no skilled assistance		Ref.	Ref.
At home with skilled assistance		0.91 (0.83–1.00)	0.92 (0.84–1.00)
At health facility		0.82 (0.78–0.86)***	0.82 (0.78–0.87)***
Birth order			
First born		Ref.	Ref.
2-3 born		1.35 (1.28–1.42)***	1.35 (1.28–1.42)***
4-5 born		1.54 (1.43–1.64)***	1.53 (1.43–1.64)***
6+ born		1.77 (1.62–1.93)***	1.76 (1.62–1.92)***
Previous birth interval			
19–35 mths		Ref.	Ref.
<19 mths		1.18 (1.10–1.26)***	1.18 (1.10–1.26)***
≥35 mths		0.79 (0.76–0.83)***	0.79 (0.76–0.83)***
Maternal age at child birth			
<20 years		Ref.	Ref.
20–24 years		0.82 (0.78–0.86)***	0.82 (0.77–0.86)***
25–29 years		0.72 (0.68–0.77)***	0.72 (0.68–0.77)***
30–34 years		0.66 (0.61–0.71)***	0.66 (0.61–0.71)***
≥35 years		0.62 (0.56–0.67)***	0.61 (0.56–0.67)***
Male child			
No		Ref.	Ref.
Yes		1.24 (1.20–1.29)***	1.24 (1.20–1.29)***
Multiple birth			
Singleton		Ref.	Ref.
Multiple birth		1.88 (1.67–2.11)***	1.90 (1.70–2.14)***
Duration of residence			
≥15 years		Ref.	Ref.
<4 years		0.99 (0.95–1.05)	0.99 (0.95–1.04)
5–14 years		0.96 (0.92–1.01)	0.97 (0.92–1.01)
Child age (yrs)		1.13 (1.11–1.15)***	1.09 (1.07–1.11)***
Breastfeeding			
Delayed		Ref.	Ref.
Early		0.97 (0.93–1.01)	0.97 (0.93–1.01)
Unknown timing		1.03 (0.95–1.13)	1.08 (0.99–1.17)
Never breastfed		1.10 (0.99–1.22)	1.12 (1.01–1.24)*
Interactions			
Slum neighborhood∗child age			1.19 (1.16–1.23)***

Note: level 2 and level 3 variables are contextual variables. Level 1 variables are compositional variables.

*P < 0.05, **P < 0.01, ***P < 0.001.
